# Utility and safety of draining pleural effusions in mechanically ventilated patients: a systematic review and meta-analysis

**DOI:** 10.1186/cc10009

**Published:** 2011-02-02

**Authors:** Ewan C Goligher, Jerome A Leis, Robert A Fowler, Ruxandra Pinto, Neill KJ Adhikari, Niall D Ferguson

**Affiliations:** 1 Interdepartmental Division of Critical Care, Mount Sinai Hospital and the University Health Network, University of Toronto, 600 University Avenue, Toronto, Ontario, M5G 1X5, Canada; 2 Department of Medicine, Mount Sinai Hospital and the University Health Network, University of Toronto, 600 University Avenue, Toronto, Ontario, M5G 1X5, Canada; 3 Department of Critical Care Medicine, Sunnybrook Health Sciences Centre, and the Interdepartmental Division of Critical Care, University of Toronto, 2075 Bayview Avenue, Toronto, Ontario, M4N 3M5, Canada; 4 Department of Medicine, Division of Respirology, Mt. Sinai Hospital and the University Health Network, and the Interdepartmental Division of Critical Care, University of Toronto, 600 University Avenue, Toronto, Ontario, M5G 1X5, Canada

## Abstract

**Introduction:**

Pleural effusions are frequently drained in mechanically ventilated patients but the benefits and risks of this procedure are not well established.

**Methods:**

We performed a literature search of multiple databases (MEDLINE, EMBASE, HEALTHSTAR, CINAHL) up to April 2010 to identify studies reporting clinical or physiological outcomes of mechanically ventilated critically ill patients who underwent drainage of pleural effusions. Studies were adjudicated for inclusion independently and in duplicate. Data on duration of ventilation and other clinical outcomes, oxygenation and lung mechanics, and adverse events were abstracted in duplicate independently.

**Results:**

Nineteen observational studies (*N *= 1,124) met selection criteria. The mean P_a_O_2_:F_i_O_2 _ratio improved by 18% (95% confidence interval (CI) 5% to 33%, *I*^*2 *^= 53.7%, five studies including 118 patients) after effusion drainage. Reported complication rates were low for pneumothorax (20 events in 14 studies including 965 patients; pooled mean 3.4%, 95% CI 1.7 to 6.5%, *I*^*2 *^= 52.5%) and hemothorax (4 events in 10 studies including 721 patients; pooled mean 1.6%, 95% CI 0.8 to 3.3%, *I*^*2 *^= 0%). The use of ultrasound guidance (either real-time or for site marking) was not associated with a statistically significant reduction in the risk of pneumothorax (OR = 0.32; 95% CI 0.08 to 1.19). Studies did not report duration of ventilation, length of stay in the intensive care unit or hospital, or mortality.

**Conclusions:**

Drainage of pleural effusions in mechanically ventilated patients appears to improve oxygenation and is safe. We found no data to either support or refute claims of beneficial effects on clinically important outcomes such as duration of ventilation or length of stay.

## Introduction

Pleural effusions are common in the critically ill, occurring in over 60% of patients in some series [1,2]. Causes are multifactorial and include heart failure, pneumonia, hypoalbuminemia, intravenous fluid administration, atelectasis and positive pressure ventilation [1-5]. However, the impact of pleural effusions on the clinical outcomes of critically ill patients is unclear. Although the presence of pleural effusion on chest radiography has been associated with a longer duration of mechanical ventilation and ICU stay, the causal relationship is unclear [2]. Data from animal studies suggest that pleural effusions reduce respiratory system compliance and increase intrapulmonary shunt with consequent hypoxemia [6-8]. In spontaneously breathing patients, drainage of large pleural effusions by thoracentesis generally produces only minor improvements in lung mechanics and oxygenation but significantly relieves dyspnea in most cases [9-17]. Complications of pleural drainage, such as pneumothorax, remain an important concern for many physicians, particularly in mechanically ventilated patients [18].

Given the uncertain benefits and risks of thoracentesis in mechanically ventilated patients, we conducted a systematic review of the literature to determine the impact of draining effusions in mechanically ventilated patients on clinical and physiologic outcomes and to ascertain the risk of serious procedural complications.

## Materials and methods

### Data sources and searches

We searched Medline (1954 to April 2010), EMBASE (1980 to April 2010), HealthStar (1966 to March 2010) and CINAHL (1990 to April 2010) using a sensitive search strategy combining MeSH headings and keywords to identify studies of critically ill, mechanically ventilated patients who underwent drainage of a pleural effusion (see Appendix). Search terms were defined *a priori *and by reviewing the MeSH terms of articles identified in preliminary literature searches. We contacted the authors of the papers identified and other opinion leaders to identify any other relevant studies. Two authors (ECG, JAL) independently reviewed the abstracts of all articles identified by the literature search and selected articles for detailed review of eligibility if either reviewer considered them potentially relevant. We also searched the bibliographies of all articles selected for detailed review and all relevant published reviews to find any other studies potentially eligible for inclusion.

### Study selection

We selected observational studies or controlled trials meeting the following inclusion criteria: (1) adult patients receiving invasive mechanical ventilation; (2) pleural effusion confirmed by any imaging modality; (3) thoracentesis or placement of a catheter or tube to drain the pleural effusion; and, (4) clinical outcomes or physiological outcomes or complications reported. Clinical outcomes included duration of mechanical ventilation (primary outcome), mortality, ICU and hospital length of stay, and new clinical management actions based on pleural fluid analysis. Physiological outcomes included changes in oxygenation (ratio of partial pressure of oxygen in systemic arterial blood (P_a_O_2_) to inspired fraction of oxygen (F_i_O_2_), alveolar-arterial gradient of P_a_O_2_, shunt fraction) and lung mechanics (peak inspiratory pressure, plateau pressure, tidal volume, respiratory rate, dynamic compliance). We recorded the occurrence of pneumothorax and hemothorax and other reported complications. We considered studies enrolling both mechanically ventilated and non-ventilated patients for inclusion if outcomes were reported separately for the mechanically ventilated subgroup. We excluded single case reports and studies of patients with pleural effusions that had absolute indications for drainage (for example, empyema, hemothorax, and so on). Each potential study was reviewed for eligibility in duplicate and independently by two authors (ECG, JAL); agreement between reviewers was assessed using Cohen's κ [19]. Disagreements were resolved by consensus and consultation with a third author (NDF) when necessary.

### Data abstraction and quality assessment

We collected data on patient demographics, admission diagnosis and severity of illness; study objective, setting, and design; ventilator settings; classification of pleural effusion (exudative vs. transudative); technique of drainage, including the use of imaging guidance, the level of training of the operator, and the type of drainage procedure performed; and outcomes. Only outcomes reported in mechanically ventilated patients were abstracted. For physiologic outcomes, we abstracted outcomes data and time of data collection before and after effusion drainage (see Additional file 1 for details [20]). One author (ECG) qualitatively assessed methodological quality based on the Newcastle-Ottawa Scale [21] and the guidelines developed by the MOOSE working group [22].

### Statistical analysis

We aggregated outcomes data at the study level and performed statistical calculations with Review Manager (RevMan) 5.0 (2009; The Cochrane Collaboration, Oxford, UK) using random-effects models [23], which incorporate both within-study and between-study variation and generally provide more conservative effect estimates when heterogeneity is present. Data were pooled using the generic inverse variance method, which weights each study by the inverse of the variance of its effect estimate; the weight is adjusted in the presence of between-study heterogeneity. We verified analyses and constructed forest plots using the R statistical package, version 2.7.2 [24]. All statistical tests were two-sided. We considered *P *< 0.05 as statistically significant in all analyses and report individual trial and summary results with 95% confidence intervals (CIs).

To conduct meta-analyses of risks of pneumothorax and hemothorax, we first converted the proportion of patients in each study with each complication to an odds. The standard error of each log odds, where odds = X/(n-X) with X = events and n-X = non-events, was calculated as $\sqrt{1/X+1/(n-X)}$. Natural log-transformed odds were pooled using the generic inverse variance method. For studies reporting zero events, we added 0.5 to both the numerator and denominator. Although values for this 'continuity correction' other than 0.5 may have superior statistical performance when comparing two treatment groups [25], previous work has shown that 0.5 gives the least biased estimator of the true log odds in a single treatment group situation [26]. The pooled log odds were converted back to a proportion. For the outcome of pneumothorax, we performed a sensitivity analysis restricting studies to those using simple thoracentesis (that is, no drain left in place). We conducted further sensitivity analyses using a Bayesian model with non-informative priors as implemented in Meta-Analyst software [27]. Each analysis used 500,000 iterations and converged. To compare complications for ultrasound-guided vs. physical landmark-guided effusion drainage, we calculated an odds ratio as exp (pooled log odds for ultrasound-guided group - pooled log odds for physical landmark-guided group) and compared the pooled log odds values using a z-test.

We report differences in P_a_O_2_:F_i_O_2 _ratio (P:F ratio) using the weighted mean of mean differences (P:F ratio after drainage - P:F ratio before drainage; a measure of absolute change) and the ratio of means (P:F ratio after drainage divided by P:F ratio before drainage; a measure of relative change) [28]. To estimate the standard errors of the mean differences as well as for the ratio of the means we assumed a correlation of 0.4 for the before and after measurements. Sensitivity analyses using alternate correlations of 0, 0.3, 0.5 and 0.8 did not change the results qualitatively. We assessed between-study statistical heterogeneity for each outcome using the *I*^*2 *^measure [29,30] and considered statistical heterogeneity to be low for *I*^*2 *^= 25 to 49%, moderate for *I*^*2 *^= 50 to 74%, and high for *I*^*2 *^>75% [30].

## Results

**Figure 1:**
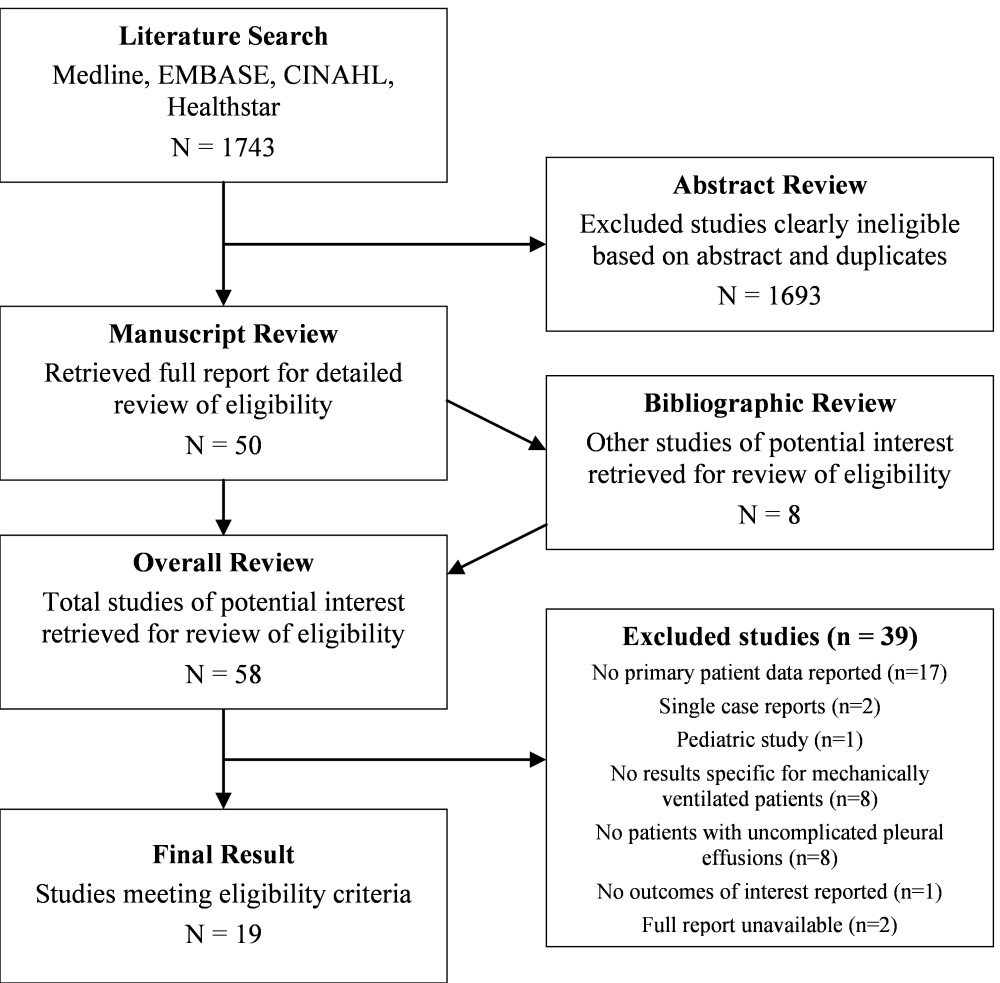
**Summary of the study selection process**.

Our search strategy identified 940 citations of interest, of which 58 reports were retrieved for full-text review (Figure 1). Nineteen studies met our selection criteria. There was excellent agreement between reviewers for study inclusion (κ = 0.88).

### Study characteristics

The 19 included studies are summarized in Table 1; the authors of three studies provided additional information [4,31,32]. Four studies measured physiological effects of pleural drainage [31,33-35]; seven studies assessed the safety of thoracentesis [36-42]; and three studies assessed the accuracy of ultrasonographic prediction of pleural effusion size [43-45]. Four studies employed real-time ultrasound guidance [32-34,46] and eight studies employed ultrasound to mark the puncture site for thoracentesis [36,38-41,43,45,47]. Twelve studies used a one-time needle/catheter thoracentsis procedure, and six studies used a temporarily secured drainage catheter or thoracostomy tube.

**Table 1:** Summary of studies included in the systematic review

Reference	Objective	Design	Population	N	Mean Age (SD)	Sex N (% Female)	Mechanical Ventilation N (%)	Intervention
Godwin 1990 [37]	Assess safety of thoracentesis in mechanically ventilated patients	Multi-centre retrospective cohort	Mechanically ventilated patients	29	Range 1 to 88 years (only 1 patient under 25 years)	Not reported	29 (100%)	Needle aspiration by medical student or resident (84%) or staff intensivist (16%) without imaging guidance
Yu 1992 [47]	Evaluate utility of chest ultrasound in diagnosis and management of critically ill patients	Single-centre prospective cohort	Critically ill patients (not all admitted to ICU^a^) with unclear findings on chest radiography	41	56 (18) years	10 (24%)	14 (34%)	Needle aspiration after puncture site marked using ultrasound guidance (performed in patients with pleural effusion on ultrasound)
McCartney 1993 [41]	Evaluate the safety of thoracentesis in mechanically ventilated patients	Single-centre prospective cohort	Patients on mechanical ventilation with a pleural effusion and a clinical indication for drainage	26	Range 19 to 92 years	Not reported	26 (100%)	Needle aspiration by staff intensivist; ultrasound employed to mark puncture site in some cases (percentage unknown)
Gervais 1997 [36]	Compare pneumothorax rates after thoracentesis between ventilated and spontaneously breathing patients	Single-centre retrospective cohort	Patients who underwent diagnostic thoracentesis in the interventional radiology suite over a four-year period. Included some pediatric patients.	434	Range 2 to 90 years	184 (42%)	90 (21%)	Needle aspiration by resident or fellow under staff supervision after marking puncture site using ultrasound guidance
Guinard 1997 [48]	Evaluate the prognostic utility of the physiologic response to a multiple component optimization strategy in ARDS^*b*^	Single-centre prospective cohort	Mechanically ventilated patients with ARDS with a lung injury score >2.5 and severe hypoxemia (mean SAPS II^*c *^46, SD 14)	36	35 (12) years	20 (56%)	36 (100%)	Drainage of pleural effusions where present (exact method not specified) along with other maneuvers to optimize gas exchange
Talmor 1998 [35]	Measure the effects of pleural fluid drainage on gas exchange and pulmonary mechanics in patients with severe respiratory failure	Single-centre prospective cohort	Surgical ICU patients on mechanical ventilation with hypoxemia unresponsive to recruitment maneuver (PEEP^*d *^20 cm H_2_O) and pleural effusions on chest radiograph (mean APACHE II^*e *^21, SD 2)	19	68 (4) years	Not reported	19 (100%)	Large-bore tube thoracostomy without imaging guidance
Lichtenstein 1999 [39]	Evaluate the safety of ultrasound-guided thoracentesis in mechanically ventilated patients	Single-centre prospective cohort	Medical ICU patients on mechanical ventilation with a pleural effusion identified by routine chest ultrasound and a clinical indication for drainage	40	64 years (SD not reported)	22 (55%)	40 (100%)	Needle aspiration by staff intensivist marking puncture site using ultrasound guidance
Fartoukh 2002 [4]	Assess the impact of routine thoracentesis on diagnosis and management	Multi-centre prospective cohort	Medical ICU patients (median SAPS II 46, range 30 to 56)	113	59 (range 42 to 68) years	54 (48%)	68 (60%)	Needle aspiration without imaging guidance
De Waele 2003 [31]	Measure the effect of drainage of pleural effusions on oxygenation	Single-centre retrospective cohort	Medical-surgical ICU patients (mean APACHE II 21, SD 8)	58	53 (19) years	19 (33%)	24 (41%)	Small-bore pigtail catheter insertion (61%) or tube thoracostomy (39%) by staff intensivist without imaging-guidance
Singh 2003 [42]	Evaluate the utility and safety of a 16-gauge catheter system for draining pleural effusions	Multi-centre prospective cohort	ICU patients with a large pleural effusion thought to contribute to respiratory impairment	10	Not reported	Not reported	8 (80%)	Small-bore catheter insertion without imaging guidance
Ahmed 2004 [33]	Measure effects of thoracentesis on hemodynamic and pulmonary physiology	Single-centre prospective cohort	Mechanically ventilated surgical ICU patients with a pulmonary artery catheter and a large pleural effusion and a clinical indication for drainage (mean APACHE II 17, SD 6)	22	63 (18) years	10 (45%)	22 (100%)	Small-bore pigtail catheter inserted under real-time ultrasound guidance
Mayo 2004 [40]	Evaluate the safety of ultrasound-guided thoracentesis in mechanically ventilated patients	Single-centre prospective cohort	Medical ICU patients on mechanical ventilation with a pleural effusion and a clinical indication for drainage	211	Not reported	Not reported	211 (100%)	Needle aspiration, small-bore pigtail catheter insertion, or large-bore tube thoracostomy by medical housestaff under staff supervision after puncture site marked using ultrasound guidance
Tu 2004 [46]	Assess the need for thoracentesis in febrile medical ICU patients and the utility of ultrasonography for diagnosing empyema	Single-centre prospective cohort	Medical ICU patients with temperature >38°C for at least eight hours and a pleural effusion on chest radiography and ultrasound	94	66 (19) years	39 (41%)	81 (86%)	Needle aspiration under real-time ultrasound guidance
Roch 2005 [44]	Evaluate the accuracy of ultrasonography to predicting size of pleural effusion	Single-centre prospective cohort	Medical-surgical ICU patients on mechanical ventilation with a clinical indication for thoracentesis	44	60 (11)	16 (36%)	44 (100%)	Large-bore tube thoracostomy without imaging guidance
Vignon 2005 [45]	Evaluate the accuracy of ultrasonography to predicting size of pleural effusion	Single-centre prospective cohort	Medical-surgical ICU patients with suspected pleural effusion based on physical examination or unexplained hypoxemia	116	60 (20) years	41 (35%)	68 (59%)	Needle aspiration after puncture site marked using ultrasound guidance
Balik 2006 [43]	Assess the utility of ultrasonography to predict pleural effusion size	Single-centre prospective cohort	Sedated and mechanically ventilated medical ICU patients with a large pleural effusion and a clinical indication for thoracentesis (mean APACHE II 20, SD 7)	81	60 (15) years	34 (42%)	81 (100%)	Needle aspiration (84%) or small-bore pigtail catheter insertion (16%) by staff intensivist after marking puncture site using ultrasound guidance
Doelken 2006 [34]	Measure the effects of thoracentesis on gas exchange and pulmonary mechanics	Single-centre prospective cohort	Mechanically ventilated patients with a large pleural effusion and a clinical indication for drainage	8	74 (20) years	5 (63%)	8 (100%)	Needle aspiration under real-time ultrasound guidance
Tu 2006 [32]	Describe the epidemiology and bacteriology of parapneumonic effusions and empyema in the ICU	Single-centre prospective cohort	Medical ICU patients with temperature >38°C for at least eight hours and a pleural effusion on chest radiography and ultrasound	175	65 (18) years	65 (37%)	148 (84%)	Needle aspiration under real-time ultrasound guidance
Liang 2009 [38]	Measure the effectiveness and safety of pigtail catheters for drainage of pleural effusions in the ICU	Single-centre retrospective cohort	Medical-surgical ICU patients with a pleural effusion who underwent pigtail catheter insertion (mean APACHE II 17, SD 7)	133	64 (15) years	40 (30%)	108 (81%)	Small-bore pigtail catheter insertion by staff intensivist after marking puncture site using ultrasound guidance

^*a*^ICU = intensive care unit.

^*b*^ARDS = acute respiratory distress syndrome.

^*c*^SAPS = Simplified Acute Physiology Score.

^*d*^PEEP = positive end-expiratory pressure.

^*e*^APACHE = acute physiology and chronic health evaluation.

The 19 included studies enrolled 1,690 patients, of which 1,124 patients received mechanical ventilation (median 40 mechanically ventilated patients per study, range 8 to 211). The mean age of enrolled patients ranged from 35 to 74 years. Of 494 patients in six studies reporting the type of effusion [4,31,34,40,42,46], 42% were classified as exudative, 55% transudative (as defined in each study), and the remaining 3% had indeterminate biochemical findings.

### Methodological quality

There were no randomized or non-randomized controlled trials of effusion drainage. Fifteen were prospective cohort studies [4,32-35,38-48] and four were retrospective cohort studies [31,36-38]. Most studies reported how patients were identified for inclusion and clearly outlined how the outcomes of pleural drainage were ascertained (see Additional file 1).

### Clinical outcomes

Only data for mechanically ventilated patients were included. Given the absence of controlled studies, the effect of pleural drainage on duration of mechanical ventilation, ICU length of stay, or hospital length of stay could not be determined. One study (*n *= 44) compared ICU length of stay between patients with pleural effusion volume drainage greater vs. less than 500 mL and found no difference [44]. Fartoukh *et al. *reported that the results of thoracentesis (*n *= 113) changed the diagnosis in 43% of patients and modified the treatment plan in 31% [4]. They found no significant reductions in duration of ICU stay or ICU mortality in patients whose management was altered by the results of thoracentesis compared to patients whose management was unchanged. Godwin *et al. *found that the results of thoracentesis affected management in 24 (75%) of 32 cases [37].

### Oxygenation

**Figure 2:**
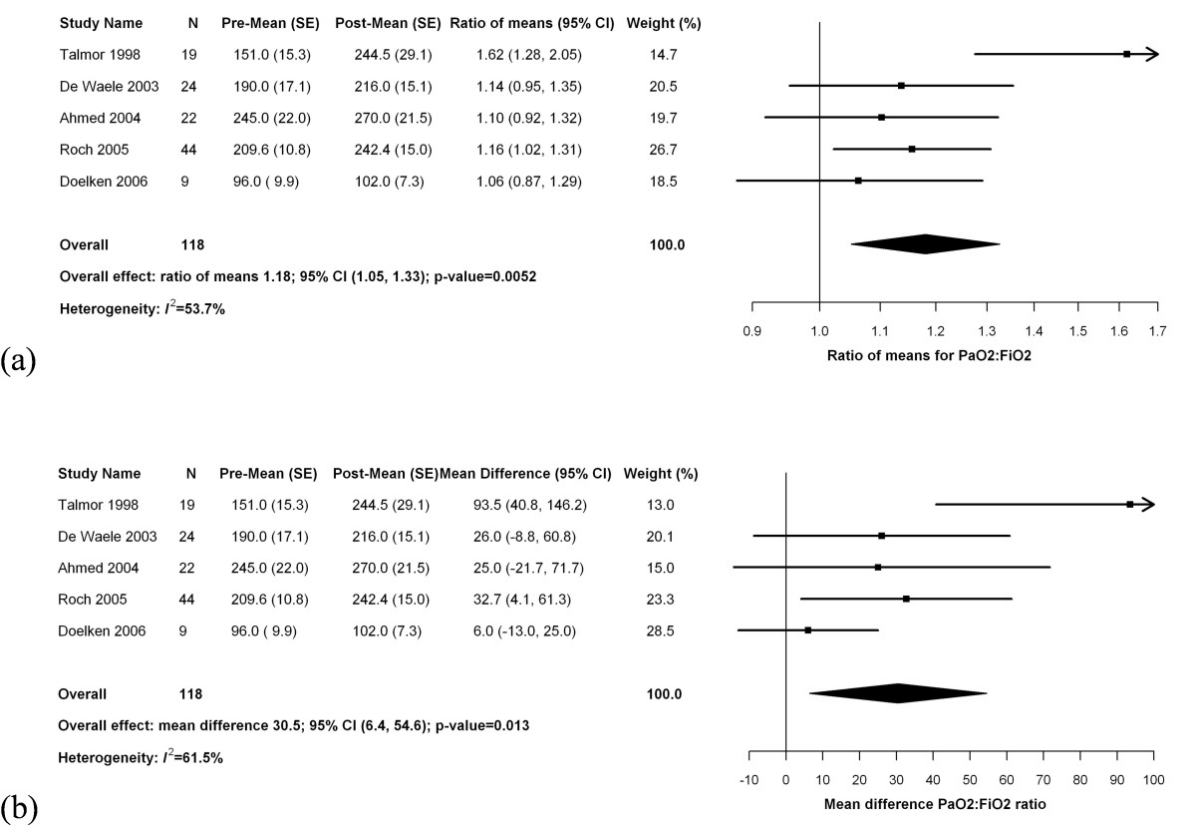
**Forest plot of meta-analysis of studies reporting change in oxygenation after pleural drainage**. P_a_O_2_:F_i_O_2 _ratios before and after thoracentesis analyzed by **(a)** relative mean difference (ratio of means) and **(b)** absolute mean difference.

Six studies described the effects of thoracentesis on oxygenation (Table 2). One study of patients with severe acute respiratory distress syndrome included thoracentesis as part of a multimodal intervention for refractory hypoxemia that also mandated diuresis, optimization of conventional ventilation, permissive hypercapnia, and adjunctive measures such as prone positioning and inhaled nitric oxide. The effect of thoracentesis alone was unclear [48]. In the remaining five studies, the timing of gas exchange measurements, volume of drainage, ventilator settings, and the measured change in oxygenation after pleural drainage varied considerably. Meta-analysis (Figure 2) demonstrated an 18% improvement in the P:F ratio after thoracentesis (95% CI 5 to 33%, *I*^*2 *^= 53.7%, five studies including 118 patients) corresponding to an increase of 31 mm Hg (95% CI 6 to 55 mm Hg, *I*^*2 *^= 61.5%, five studies including 118 patients).

**Table 2:** Summary of studies of oxygenation after thoracentesis in mechanically ventilated patients

Study	**N on MV**^*a*^	**PEEP**^***b***^** (cm H**_**2**_**O)**	Volume Drained (mean ± SD)	Time of Outcome Measurement	Variable	**Outcome**^*d*^		
						
						Before	After	*P*-value
Ahmed 2004	22	Not reported	1,262 ± 762 mL (Initial drainage)	<1 hour before and after drainage	P_a_O_2_:F_i_O_2_	245 ± 103	270 ± 101	0.31^*c*^
					A-a Gradient	236 ± 170	211 ± 153	0.52^*c*^
					Shunt Fraction	26.6 ± 15.1	21.0 ± 7.8	0.03

De Waele 2003	24	Not reported	1,077 mL (SD not reported) (Over first 24 hours)	Before and 24 hours after drainage	P_a_O_2_:F_i_O_2_	190 ± 84	216 ± 74	0.16^*c*^

Doelken 2006	9	0	1,575 ± 450 mL (Initial drainage)	Immediately before and after procedure	P_a_O_2_:F_i_O_2_^*e*^	96 ± 29.7	102 ± 21.9	0.37
					A-a Gradient	226 ± 99.6	217 ± 85.2	0.34

Guinard 1997	36	12 ± 3	n/a	6 to 12 hours post-optimization procedure	Predefined gas exchange response ^*d*^		53% responded	

Roch 2005	44	6 ± 2	730 ± 440 mL (first three hours)	Before and 12 hours after drainage	P_a_O_2_:F_i_O_2 _(effusion <500 mL) (*N *= 20)	214 ± 83	232 ± 110	0.47^*c*^
					P_a_O_2_:F_i_O_2 _(effusion >500 mL) (*N *= 24)	206 ± 62	251 ± 91	<0.01

Talmor 1998	19	17 ± 1	863 ± 164 mL (first eight hours)	Immediately before and 24 hours after drainage	P_a_O_2_:F_i_O_2_	151.0 ± 66.7	244.5 ± 126.8	<0.0001

^*a*^MV, mechanical ventilation.

^*b*^PEEP, positive end-expiratory pressure.

^*c *^*P*-value not provided in the original paper; we calculated the *P*-value based on the data provided, assuming a correlation between the before and after measurements of 0.4

^*d *^P_a_O_2 _>100 mm Hg on F_i_O_2 _1.0 for at least six hours.

^*a *^Values are reported as mean **± **standard deviation.

^*e *^Original paper reported P_a_O_2 _but all patients were on F_i_O_2 _1.0; we calculated P_a_O_2_:F_i_O_2 _from these data.

Some studies identified possible predictors of improved oxygenation after thoracentesis. Roch *et al. *(*n *= 44) found that the increase in the P:F ratio correlated with the effusion volume drained (r = 0.5, *P *= 0.01) in the subgroup of patients with pleural effusions greater than 500 mL in size (*n *= 24). Conversely, Talmor *et al. *(*n *= 19) found no relationship between oxygenation response and the drained volume. In a multivariate analysis by De Waele *et al. *(*n *= 24), a P:F ratio less than 180 mm Hg was the sole independent predictor of improved P:F ratio after thoracentesis [31].

### Lung mechanics

Three studies reported on the association of thoracentesis with changes in lung mechanics (Table 3). Talmor *et al. *(*n *= 19) reported a 30% increase in dynamic compliance immediately after the procedure and Doelken *et al. *(*n *= 9) reported a trend toward increased dynamic compliance. Doelken *et al. *also found a statistically significant reduction in the work of inflation per cycle (calculated by integration of the pressure-time curve) after thoracentesis. Ahmed *et al. *(*n *= 22) observed a reduction in the respiratory rate after thoracentesis but there was no significant change in lung mechanics.

**Table 3:** Summary of studies of pulmonary mechanics after thoracentesis in mechanically ventilated patients

Study	Proportion Mechanically Ventilated	N	Time of Outcome Measurement	Variable	**Outcome**^***a***^		
					
					Before	After	*P*-value
Ahmed 2004	100%	22	<1 hour before and after thoracentesis	Peak inspiratory pressure (cm H_2_O)	34.9 ± 8.4	35.9 ± 12.5	0.64^*b*^
				Respiratory rate	19.4 ± 6.5	15.5 ± 6.3	0.03

Doelken 2006	100%	9	Immediately before and after procedure	Peak inspiratory pressure (cm H_2_O)	43.8 ± 13.7	40.8 ± 10.6	0.08
				Plateau pressure (cm H_2_O)	20.0 ± 9.0	17.8 ± 5.6	0.19
				Dynamic compliance (L/cm H_2_O)	14.5 ± 5.3	15.2 ± 5.0	0.12
				Ventilator work per cycle (Joules)	3.42 ± 1.05	2.99 ± 0.81	0.01

Talmor 1998	100%	19	Immediately before and after procedure	Peak inspiratory pressure (cm H_2_O)	44.3 ± 13.9	42.9 ± 18.7	0.74^*b*^
				Dynamic compliance (L/cm H_2_O)	27.1 ± 15.3	35.7 ± 30.5	< 0.05

^*a *^Values are reported as mean **± **standard deviation.

^*b *^*P*-value not provided in the original paper; we calculated the *P*-value based on the data provided, assuming a correlation between the before and after measurements of 0.4.

### Complications

**Figure 3:**
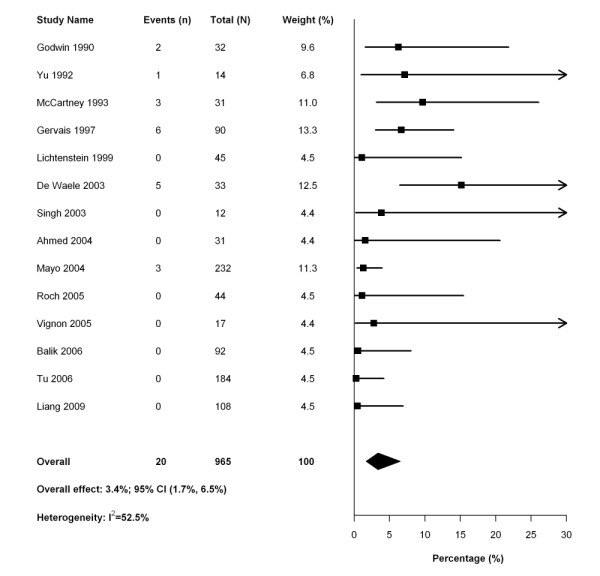
**Forest plot of meta-analysis of studies reporting the rate of pneumothorax after pleural drainage**.

**Figure 4:**
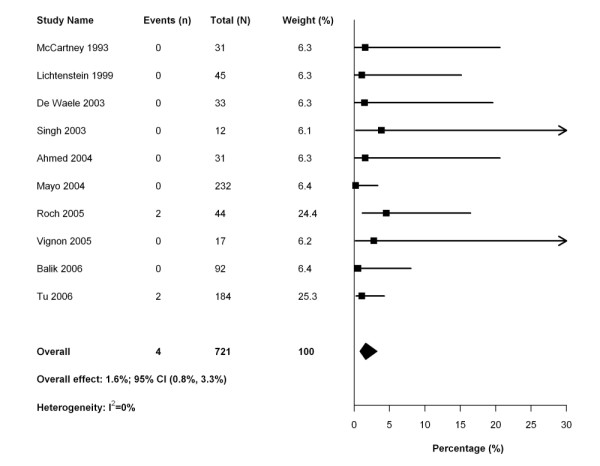
**Forest plot of meta-analysis of studies reporting the rate of hemothorax after pleural drainage**.

Sixteen studies reported complications associated with thoracentesis (Table 4), and all but one [33] prespecified detection of complications in the study protocol. One study [32] included complication data from an earlier study that included some of the same patients [46]; the earlier study was removed from further analysis of complications. One study [4] did not report the number of procedures performed in mechanically ventilated patients and, therefore, could not be included in this calculation. The pooled risk of post-thoracentesis pneumothorax was 3.4% (95% CI 1.7 to 6.5%; 20 events in 14 studies including 965 patients) (Figure 3). After excluding studies that employed a temporary drain to perform the drainage procedure, the pooled risk of pneumothorax was 4.3% (95% CI 2.1 to 8.7%; 12 events in 8 studies including 496 patients). The pooled risk of hemothorax was 1.6% (95% CI 0.8 to 3.3%; 4 events in 10 studies with 721 patients) (Figure 4). The use of ultrasound guidance was not associated with a reduction in pneumothorax (OR 0.32; 95% CI 0.08 to 1.19). Sensitivity analyses using Bayesian models estimated an even lower risk of complications (pneumothorax: 1.3%, 95% credible interval 0.2% to 3.3%; hemothorax 0.5%, 95% credible interval 0% to 1.2%).

**Table 4:** Thoracentesis complication rates in mechanically ventilated patients

Reference	Operator training	Ultrasound guidance	**Systematic detection **^***a***^	# Procedures in MV patients	Pneumothorax rate	Hemothorax rate	Additional findings
Godwin 1990	Student or resident (84%) or staff intensivist (16%)	None	Yes	32	6.3%	n/a ^*b*^	The pneumothoraces occurred after procedures performed by house staff No tension pneumothoraces
Yu 1992	Not specified	Puncture site marked	Yes	14	7.1%	n/a	
McCartney 1993	Staff intensivist	Puncture site marked in some cases	Yes	31	9.7%	0%	No tension pneumothoraces
Gervais 1997	Resident or fellow	Puncture site marked	Yes	90	6.7%	n/a	Only 1% of non-MV patients had pneumothorax (difference in rates was statistically significant) Only two of ten pneumothoraces required chest tubes (rest too small)
Lichtenstein 1999	Staff intensivist	Puncture site marked	Yes	45	0%	0%	
Fartoukh 2002	Not reported	None	Yes	Unknown	n/a	n/a	Five of six reported pneumothoraces occurred in patients on MV
De Waele 2003	Staff intensivist	None	Yes	33	15%	0%	nine pneumothoraces in all patients hemothorax
Singh 2003	Not specified	None	Yes	12	0%	0%	
Ahmed 2004	Not reported	Real-time guidance	No	31	0.0%	0%	
Mayo 2004	Resident or fellow	Puncture site marked	Yes	232	1.3%	0%	No tension pneumothoraces
Tu 2004	Not specified	Real-time guidance	Yes	Unknown	0%	n/a	No pneumothoraces in all patients two hemothoraces in all patients (Data included in Tu 2006)
Roch 2005	Not specified	None	Yes	44	0%	4.5%	
Vignon 2005	Not specified	Puncture site marked	Yes	17	0%	0%	Pneumothorax data available only on 17 MV patients (unknown how many other procedures were done on patients on MV)
Balik 2006	Staff intensivist	Puncture site marked	Yes	92	0.0%	0%	
Tu 2006	Not specified	Real-time guidance	Yes	184	0%	1.1%	
Liang 2009	Staff intensivist	Puncture site marked	Yes	108	0%	n/a	one hemothorax in all patients No pneumothoraces in non-MV patients three subcutaneous hematomas four infections related to drainage seven kinked catheters

^*a *^Protocol included pre-specified detection of complications of procedure including either chest radiography or chest ultrasound.

^*b *^n/a, not available; that is, not reported in the paper or, if reported, the rate is not specific to patients on mechanical ventilation.

## Discussion

This systematic review demonstrates that pleural drainage in mechanically ventilated patients is associated with improved oxygenation and a reassuringly low risk of serious peri-procedural complications. There was some data to suggest that routine diagnostic thoracentesis may alter the diagnosis or management of this patient population. However, there were no data on the impact of pleural drainage on duration of mechanical ventilation, our primary outcome of interest. Furthermore, there were no controlled studies of thoracentesis for any clinical or physiological end-point. We conclude that there is no definite evidence to recommend for or against draining pleural effusions in mechanically ventilated patients to improve major clinical outcomes including mortality, duration of mechanical ventilation, or length of ICU or hospital stay.

Studies of the effect of effusion drainage on oxygenation report heterogeneous findings; these differences may be attributable to systematic variation in severity of pre-existing hypoxemia, lung and chest wall compliance, positive-end expiratory pressure settings, pleural effusion volume, and timing of observations. Studies of non-ventilated patients have documented relatively minor improvements in oxygenation after effusion drainage [9,12,49,50]. Small or moderate-sized effusions do not ordinarily cause significant hypoxemia because most (75 to 80%) of the effusion volume is accommodated by the compliant chest wall and flattening of the diaphragm [6,10,14,51]. When chest wall compliance is reduced or the pleural effusion is large, effusions cause hypoxemia by collapsing adjacent lung with resultant physiologic shunt [12,49]. Drainage of pleural effusions may improve hypoxemia by allowing re-expansion of collapsed lung, which proceeds variably over the subsequent 24 hours [14] and may continue for several weeks [13,52]. In our review, one study [35] found significant improvement in oxygenation with thoracentesis by pre-selecting patients for study whose hypoxemia was refractory to high positive end-expiratory pressure (PEEP). This approach may have identified patients with reduced chest wall or abdominal compliance whose oxygenation would be predicted to improve after effusion drainage [3]. In addition, the application of high PEEP may have promoted rapid recruitment of collapsed lung after effusion drainage.

A number of questions related to the impact of pleural effusion drainage on gas exchange remain unaddressed. These include the notion of the minimally important drainage volume and the use of maneuvers to re-expand previously collapsed lung after effusion drainage such as the application of PEEP. Also, it is unclear whether the degree of improvement in oxygenation after drainage depends on the severity of baseline hypoxemia or the total amount of fluid removed. In our systematic review, we did not perform meta-regression to assess the effect of either variable on improvement in oxygenation because of the limited number of studies, differences among studies in ventilator settings (making the interpretation of baseline hypoxemia difficult), and risk of ecological bias [53], since study-level meta-regression cannot determine whether patients *within *each study with more severe hypoxemia or more fluid drained benefited more.

Drainage of pleural effusions is sometimes proposed to accelerate weaning from mechanical ventilation. The underlying assumption is that pleural effusions decrease respiratory system compliance; drainage of effusions may therefore improve respiratory system mechanics and reduce ventilatory load. This review did not identify studies to strongly support or refute this hypothesis. Two uncontrolled studies [34,35] found relatively minor improvements in measures of compliance after effusion drainage, but it is unclear whether these changes would accelerate liberation from the ventilator. Data from spontaneously breathing patients show that drainage of effusions resulted in small improvements in lung volumes and static compliance [9-11,13-15,17], which would not likely explain the immediate relief of dyspnea reported by many patients.

Alternatively, draining pleural effusions may reduce the work of breathing by improving the mechanics of the diaphragm. Multiple authors have reported mechanical abnormalities of the diaphragm in the presence of pleural effusions including diaphragmatic inversion and paradoxical motion [11,17,54-56]. In a study of spontaneously breathing patients, Estenne *et al. *observed a marked increase in maximal inspiratory pressure immediately after thoracentesis [10] suggesting that diaphragmatic function might be impaired in the presence of a pleural effusion. They attributed the significant relief of dyspnea reported by patients after thoracentesis to improved diaphragm mechanics. In a recent study, the presence of paradoxical motion of the diaphragm in patients with pleural effusions predicted significantly greater improvements in dyspnea after thoracentesis [17]. Further research is necessary to replicate these findings in mechanically ventilated patients and to measure the potential benefit of pleural effusion drainage on duration of ventilation and other relevant clinical outcomes.

Clinicians may hesitate to perform thoracentesis in mechanically ventilated patients due to the risk of complications, particularly pneumothorax. This systematic review included studies that varied in setting, technique, use of ultrasound guidance and operator experience and found a low risk of complications in mechanically ventilated patients; the risk of pneumothorax was similar when studies were restricted to those performing simple thoracentesis with no drainage tube left in place. Sensitivity analysis using Bayesian methods found an even lower rate of complications than traditional meta-analysis. However, the true risk of complications may be higher outside of a study. We did not detect a reduction in complications associated with the use of periprocedural ultrasound guidance. A recent review of pneumothorax following thoracentesis among 24 studies of mostly spontaneously breathing patients found an overall rate of pneumothorax of 6.0% (95% CI 4.6% to 7.8%), of whom one-third required thoracostomy tube placement [57]. Ultrasound guidance was associated with a reduced risk of pneumothorax in that study. Although the risk of pneumothorax was non-significantly higher among patients receiving mechanical ventilation in that review, we found a lower rate among studies restricted to mechanically ventilated patients. This may be (1) because in our review drainage methods frequently included temporarily secured drainage catheters or thoracostomy tubes (that eliminate most pneumothoraces if they occur); (2) mechanically ventilated patients may be sedated for the procedure allowing for optimal positioning and reducing patient movement and therefore a lower risk of lung puncture; or (3) possibly due to other differences in operator characteristics or approach for patients receiving mechanical ventilation. Additionally, while we included all studies in the published meta-analysis that provided specific data on mechanically ventilated patients, we also incorporated data from several additional studies [4,31-33,38,41-47].

Strengths of this review include a broad literature search supplemented by contact with primary study investigators, consideration of a comprehensive set of outcomes, and consideration of alternate analytical approaches in sensitivity analyses. There are important limitations to this review related to the absence of controlled trials of pleural effusion drainage and lack of data on ventilator settings before and after drainage in some studies, which limited inferences regarding the effect of drainage on lung mechanics.

## Conclusions

In summary, our systematic review did not identify any controlled studies of pleural effusion drainage in mechanically ventilated patients. Limited data suggest that pleural drainage is safe, may improve oxygenation, and under certain conditions may improve respiratory mechanics. We were unable to identify any evidence to support or refute the use of pleural drainage to promote liberation from mechanical ventilation. Further research is necessary and should focus on clarifying the physiological effects of pleural fluid drainage, the impact of the procedure on important clinical outcomes, the conditions under which a therapeutic response may be achieved, and the characteristics of those patients most likely to benefit from the procedure.

## Key messages

• Pleural drainage is associated with minor improvements in oxygenation and lung mechanics.

• The complication rate from pleural drainage is very low. In our meta-analysis, the risk of post-thoracentesis pneumothorax was 3.4% (95% CI 1.7 to 6.5%; 20 events in 14 studies including 965 patients) and the pooled risk of hemothorax was 1.6% (95% CI 0.8 to 3.3%; 4 events in 10 studies including 721 patients).

• We could not find any studies reporting duration of ventilation or other clinically relevant ICU outcomes and further investigation is required to evaluate the benefit of pleural drainage in terms of liberation from mechanical ventilation.

## Abbreviations

APACHE: Acute Physiology And Chronic Health Evaluation; ARDS: Acute Respiratory Distress Syndrome; MV: mechanical ventilation; PEEP: Positive End-Expiratory Pressure; P:F ratio: P_a_O_2_:F_i_O_2 _ratio; SAPS: Simplified Acute Physiology Score.

## Competing interests

The authors declare that they have no competing interests.

## Authors' contributions

EG participated in study design, data collection, data analysis and manuscript preparation. JL participated in data collection and manuscript preparation. RF participated in study design, data analysis and manuscript preparation. RP participated in data analysis and manuscript preparation. NA participated in data analysis and manuscript preparation. NF participated in study design and manuscript preparation.

## Supplementary Material

Additional file 1 **Online appendix**. Provides more in-depth details on literature search methods and results, data abstraction, quality assessment, and statistical analysis. Click here for file
