# Supplementary material for: Utility and safety of draining pleural effusions in mechanically ventilated patients: a systematic review and meta-analysis
Source: Crit Care. 2011 Feb 2;15(1):R46. doi: 10.1186/cc10009 (PMC3221976; doi:10.1186/cc10009)
Supplement: Additional file 1 — Online appendix. Provides more in-depth details on literature search methods and results, data abstraction, quality assessment, and statistical analysis. [file cc10009-S1.DOC]

**Online Data Supplement**

A Systematic Review of the Utility and Safety of Draining Pleural Effusions in Mechanically Ventilated Patients

Ewan C. Goligher, Jerome A. Leis, Robert A. Fowler,

Ruxandra Pinto, Neill K. J. Adhikari, and Niall D. Ferguson

This supplement provides details of the search strategy and statistical analysis for this systematic review and provides a list of included studies together with our assessment of their methodological quality.

**Methods**

**Data Sources and Searches**

We searched Medline (1954 to April 2010), EMBASE (1980 to April 2010), HealthStar (1966 – March 2010) and CINAHL (1990 to April 2010) using a sensitive search strategy combining MeSH headings and keywords to identify studies of critically ill, mechanically ventilated patients who underwent drainage of a pleural effusion (see Search Strategy below). Search terms were defined a priori and by reviewing the MeSH terms of articles identified in preliminary literature searches. We contacted the authors of the papers identified and other international opinion leaders to identify any other relevant studies. Two authors (EG, JL) independently reviewed the abstracts of all articles identified by the literature search and selected articles for detailed review of eligibility if either reviewer considered them potentially relevant. We also searched the bibliographies of all articles selected for detailed review and all relevant published reviews to find any other studies potentially eligible for inclusion.

**Study Selection**

Each potential study was reviewed in duplicate for eligibility by two authors (EG, JL) working independently. Disagreements were resolved by consensus or consultation with a third author (NDF) when necessary. We selected studies meeting the following inclusion criteria: (1) studies of adult patients receiving invasive mechanical ventilation, (2) patients had a pleural effusion confirmed by imaging, (3) patients underwent a drainage procedure of any type for their pleural effusion, and (4) the study reported any of the following outcomes specifically for mechanically ventilated patients: mortality, ICU length-of-stay, hospital length-of stay, duration of mechanical ventilation, gas exchange, lung mechanics, hemodynamics, change in management, or complications. We excluded single case reports and studies of patients with pleural effusions that were presumed to be complicated prior to drainage (i.e. pre-existing empyema or hemothorax).

**Data Abstraction**

We collected demographic data, information on study objective, setting, and design, ventilator settings, and information on the classification of pleural effusion (exudative vs. transudative). We recorded the technique of drainage, including the use of imaging guidance, the level of training of the operator, and the type of drainage procedure performed. We collected reported outcomes including mortality, ICU length of stay, duration of mechanical ventilation, and hospital length of stay. We recorded means, standard deviations and statistical significance of measures of gas exchange (PaO_2_:FiO_2_ ratio, A-a gradient, shunt fraction), lung mechanics (peak inspiratory pressure, plateau pressure, tidal volume, respiratory rate, dynamic compliance), and the time timing of measurement before and after the drainage procedure where reported. The frequency of alterations in diagnosis or management based on pleural fluid analysis was documented. Rates of pneumothorax, hemothorax, and other reported complications were recorded. Only reported outcomes and complications specific for mechanically ventilated patients were recorded. In studies including both ventilated and non-ventilated patients, we attempted to extract results specific to ventilated patients; if this was not possible, the results were not included in the analysis.

**Methodological Quality**

Each study was assessed qualitatively for methodological quality using a system based on the Newcastle Ottawa Scale for the assessment of observational studies [21] and the guidelines developed by the MOOSE working group [22]. We reviewed each study for information on sampling method, presence of a control group, comparability of a control group if included, means of ascertaining cases of pleural drainage (in retrospective studies), means of ascertaining outcomes (in retrospective and prospective studies), adequacy of duration of follow-up, and rates of loss to follow-up. Data were tabulated to display overall methodological quality (see Table 1 of this Appendix).

**Statistical Analysis**

We aggregated outcomes data at the study level and performed statistical calculations with Review Manager (RevMan) 5.0 (2009; The Cochrane Collaboration, Oxford, UK) using random-effects models which incorporate both within-study and between-study variation and generally provide more conservative treatment estimates when heterogeneity is present. We verified analyses and constructed forest plots using the R statistical package, version 2.7.2 [24]. All statistical tests were two sided. We considered p<0.05 as statistically significant in all analyses and report individual trial and summary results with 95% confidence intervals (CIs).

To conduct meta-analyses of risks of pneumothorax and hemothorax, we first converted the proportion of patients in each study with each complication to an odds. The standard error of each log odds, where odds = X/(n-X) with X = events and n-X = non-events, was calculated as $\sqrt{1/X+1/(n-X)}$. Natural log-transformed odds were pooled using the generic inverse variance method. For studies reporting zero events, we added 0.5 to both the numerator and denominator. Although values for this ‘continuity correction’ other than 0.5 may have superior statistical performance when comparing 2 treatment groups [25], previous work has shown that 0.5 gives the least biased estimator of the true log odds in a single treatment group situation [26]. The pooled log odds was converted back to a proportion. We conducted sensitivity analyses using a Bayesian model with non-informative priors as implemented in Meta-Analyst software [20]. Each analysis used 500,000 iterations and converged. To compare complications for ultrasound-guided vs. physical landmark-guided effusion drainage, we calculated an odds ratio as (pooled log odds for ultrasound-guided group - pooled log odds for physical landmark-guided group) and compared using a z-test.

We report differences in PaO2:FiO2 ratio (P:F ratio) using the weighted mean of mean differences (P:F ratio after drainage – P:F ratio before drainage; a measure of absolute change) and the ratio of means (P:F ratio after drainage divided by P:F ratio before drainage; a measure of relative change) [28]. To estimate the standard errors of the mean differences as well as for the ratio of the means we assumed a correlation of 0.4 for the before and after measurements. Sensitivity analyses using alternate correlations of 0, 0.3, 0.5 and 0.8 did not change the results qualitatively. We assessed between-study statistical heterogeneity for each outcome using the I^2^ measure [29, 30] and considered statistical heterogeneity to be low for I^2^ = 25–49%, moderate for I^2^ = 50–74%, and high for I^2^ > 75% (8).

**Search Strategy**

**Database: Ovid MEDLINE(R) <1950 to April Week 1 2010> Search Strategy:**

--------------------------------------------------------------------------------

1 exp Intensive Care Units/ or exp Intensive Care/ or exp Critical Care/ (72404)

2 exp Critical Illness/ (10900)

3 exp Respiratory Insufficiency/ (44678)

4 exp Respiration, Artificial/ (49276)

5 exp Positive-Pressure Respiration/ (16790)

6 mechanical ventilation.mp. (18169)

7 exp Thoracostomy/ (871)

8 exp Chest Tubes/ (1562)

9 thoracentesis.mp. (924)

10 thoracocentesis.mp. (401)

11 pleural effusion.mp. (18574)

12 exp pleural effusion/ (14010)

13 or/1-6 (162072)

14 or/7-12 (20724)

15 13 and 14 (773)

**Database: EMBASE <1980 to 2010 Week 15>**

Search Strategy:

--------------------------------------------------------------------------------

1 exp Intensive Care/ (220231)

2 exp critical care/ (220231)

3 exp Intensive Care Unit/ (34442)

4 exp Critical Illness/ (13699)

5 exp Respiratory Failure/ or exp Acute Respiratory Failure/ or exp Intermittent Positive Pressure Ventilation/ or exp Artificial Ventilation/ (80949)

6 chest tube.mp. (2362)

7 thoracocentesis.mp. (2650)

8 exp pleura effusion/ (15718)

9 pleural effusion.mp. (8137)

10 thoracentesis.mp. (810)

11 exp thoracocentesis/ (2566)

12 or/1-5 (265045)

13 or/8-9 (17622)

14 thoracostomy.mp. or exp Thorax Drainage/ (4241)

15 or/6-7,10-11,14 (8127)

16 and/12-13,15 (394)

17 limit 16 to (adolescent <13 to 17 years> or adult <18 to 64 years> or aged <65+ years>) (226)

**Database: Ovid Healthstar <1966 to March 2010> Search Strategy:**

--------------------------------------------------------------------------------

1 exp Intensive Care/ (13737)

2 exp critical care/ (31793)

3 exp Critical Illness/ (10476)

4 exp Intensive Care Units/ (40639)

5 exp Respiratory Insufficiency/ (25217)

6 exp Respiration, Artificial/ (33597)

7 exp Positive-Pressure Respiration/ (10980)

8 mechanical ventilation.mp. (13457)

9 or/1-8 (122720)

10 exp Pleural Effusion/ (6948)

11 pleural effusion.mp. (9923)

12 exp Thoracostomy/ (756)

13 thoracentesis.mp. (620)

14 thoracocentesis.mp. (237)

15 exp Chest Tubes/ (1638)

16 or/10-15 (11885)

17 9 and 16 (607)

**CINAHL** **(Ebsco) (1990 – April 2010)**

(("intensive care") or (MH "Intensive Care Units+") or (MH "Critical Care+") or (MH "Respiratory Insufficiency+") or (MH "Respiration, Artificial+") or (MH "Positive-Pressure Respiration+") or ("mechanical ventilation")) AND ((MH "Thoracostomy+") or (MH "Chest Tubes+") or ("thoracentesis") or ("thoracocentesis") or ("pleural effusion") or (MH "Pleural Effusion+")).

137 Results

Table 1. Methodological Quality of Included Studies

| **Reference** | **Representativeness of Exposed Cohort** | **Selection of Non-Exposed Cohort** | **Ascertainment of Exposure** | **Comparability of Controls** | **Ascertainment of Outcome** | **Follow-Up Adequate to Allow Outcome to Occur** | **Loss to Follow-Up** |
| --- | --- | --- | --- | --- | --- | --- | --- |
| **Godwin 1990** [37] | Consecutive sampling | No control group | Retrospective Chart Review | No control | Retrospective Chart Review | Yes | None reported |
| **Yu 1992** [47] | Biased sampling | No control group | Prospective Protocol | No control | Prospective Protocol | Yes | None reported |
| **McCartney 1993** [41] | Consecutive sampling | No control group | Retrospective Chart Review | No control | Retrospective Chart Review | Yes | None reported |
| **Gervais 1997** [36] | Consecutive sampling | No control group | Retrospective Chart Review | No control | Retrospective Chart Review | Yes | None reported |
| **Guinard 1997** [48] | Consecutive sampling | No control group | Prospective Protocol | No control | Prospective Protocol | Yes | None reported |
| **Talmor 1998** [35] | Selection not reported | No control group | Prospective Protocol | No control | Prospective Protocol | Yes | None reported |
| **Lichtenstein 1999** [39] | Consecutive sampling | No control group | Prospective Protocol | No control | Prospective Protocol | Yes | None reported |
| **Fartoukh 2002** [4] | Consecutive sampling | No control group | Prospective Protocol | No control | Prospective Protocol | Yes | None reported |
| **De Waele 2003** [31] | Selection not reported | No control group | Retrospective Chart Review | No control | Retrospective Chart Review | Yes | None reported |
| **Singh 2003** [42] | Selection not reported | No control group | Prospective Protocol | No control | Prospective Protocol | Yes | None reported |
| **Ahmed 2004** [33] | Consecutive sampling | No control group | Prospective Protocol | No control | Prospective Protocol | Yes | None reported |
| **Mayo 2004** [40] | Consecutive sampling | No control group | Prospective Protocol | No control | Prospective Protocol | Yes | None reported |
| **Tu 2004** [46] | Consecutive sampling | No control group | Prospective Protocol | No control | Prospective Protocol | Yes | None reported |
| **Roch 2005** [44] | Consecutive sampling | No control group | Prospective Protocol | No control | Prospective Protocol | Yes | None reported |
| **Vignon 2005** [46] | Selection not reported | No control group | Prospective Protocol | No control | Prospective Protocol | Yes | None reported |
| **Balik 2006** [43] | Selection not reported | No control group | Prospective Protocol | No control | Prospective Protocol | Yes | None reported |
| **Doelken 2006** [34] | Selection not reported | No control group | Prospective Protocol | No control | Prospective Protocol | Yes | None reported |
| **Tu 2006** [32] | Consecutive sampling | No control group | Prospective Protocol | No control | Prospective Protocol | Yes | None reported |
| **Liang 2009** [38] | Selection not reported | No control group | Retrospective Chart Review | No control | Retrospective Chart Review | Yes | None reported |
